# Identification and characterization of pancreatic infections in severe and critical acute pancreatitis patients using *16S rRNA* gene next generation sequencing

**DOI:** 10.3389/fmicb.2023.1185216

**Published:** 2023-06-14

**Authors:** Ning Sun, Yong Chen, Jiaxun Zhang, Jin Cao, Hongjuan Huang, Jie Wang, Wentao Guo, Xiaojun Li

**Affiliations:** ^1^Department of Clinical Laboratory Science, Jinling Hospital, Affiliated Hospital of Medical School, Nanjing University, Nanjing, China; ^2^Clinical Medicine Research Center, The Affiliated Suqian First People’s Hospital of Nanjing Medical University, Suqian, China; ^3^Department of Microbiology and Immunology, College of Basic Medicine, Guangdong Medical University, Dongguan, China

**Keywords:** *16S rRNA* NGS, severe acute pancreatitis, critical acute pancreatitis, infected pancreatic necrosis, bacterial profile

## Abstract

**Objectives:**

This study aimed to identify the bacterial composition in the pancreatic fluid of severe and critical acute pancreatitis (SAP and CAP) patients.

**Methods:**

A total of 78 pancreatic fluid samples were collected from 56 SAP and CAP patients and analyze using aerobic culture and *16S rRNA* gene next-generation sequencing. The clinical data of the patients were obtained from the electronic medical records.

**Results:**

Among the total 78 samples, *16S rRNA* gene NGS identified a total of 660 bacterial taxa, belonging to 216 species in 123 genera. The dominant aerobic bacteria included *Klebsiella pneumoniae*, *Acinetobacter baumannii*, and *Enterococcus faecium*, while the dominant anaerobic bacteria included *Bacteroides*, *Dialister invisus*, and *Olsenella uli*. As compared to aerobic culturing, 95.96% (95/99) of the aerobic cultured bacteria were detected using the *16S rRNA* gene NGS.

**Conclusion:**

The pancreatic infections in SAP and CAP patients might originate not only from the gut but also from the oral cavity and airways as well as related environments. Dynamic analysis of bacterial profile and abundance showed that some bacteria with low abundance might become the main pathogenic bacteria. There were no significant differences in the bacterial diversity between SAP and CAP.

## 1. Introduction

Acute pancreatitis (AP) is a common disease with various clinical emergency courses (Fritz et al., 2010; Dellinger et al., 2012; Banks et al., 2013). Most of the AP cases are mild and self-limited within a week. However, 15% of the cases are exacerbated to a higher level of severity known as severe or critical AP (SAP or CAP), which is associated with local complications and/or organ failure (Dellinger et al., 2012; Banks et al., 2013). Numerous studies confirmed that organ failure and infected pancreatic necrosis (IPN) were the determinants of mortality in AP patients (Dellinger et al., 2012; Banks et al., 2013). Organ failure emerges in the early and/or late phase of AP and can be assessed using a modified Marshall scoring system. Peripancreatic infections and IPN are rare during the first week (Banks et al., 2013). However, IPNs, especially multidrug-resistant microbial infections, are associated with adverse clinical outcomes and increased mortality (Ning et al., 2019; Jiang et al., 2020). Depending on the presence and absence of organ failure and local complications, the severity of AP is classified into 4 categories based on determinants, including mild, moderate, severe, and critical AP (Dellinger et al., 2012). SAP is characterized by the presence of either IPN or persistent organ failure, while CAP is defined by the presence of both IPN and persistent organ failure. Sometimes, there is also a combination of SAP and CAP (Banks et al., 2013). Secondary infection in pancreatic necrosis can cause the development of sepsis associated with fatal outcomes, and statistical analysis indicated that the patients with IPN had a significantly higher risk of death as compared to those with no IPN (Fritz et al., 2010; Petrov et al., 2010). In summary, IPN is strongly associated with the prognosis of SAP and CAP.

Generally, bacterial translocation is considered the main infection mechanism of IPN (Fritz et al., 2010). During the early phase, pancreatic inflammation can activate cytokine cascades, resulting in systemic inflammatory response syndrome and subsequently altering intestinal permeability and motility, which leads to bacterial translocation (Liang et al., 2021). Animal studies have shown that gut microbiota can cross the mucosal barrier, reaching the pancreas or other organs in different sites (Fritz et al., 2010). In clinical microbiological laboratories, pathogenic bacteria are identified and classified using culturing. A large number of bacterial species were isolated from the pancreatic drainage fluid of SAP patients using culture (Brook and Frazier, 1996; Fan et al., 2020), among which, the predominant species included *Escherichia coli*, *Klebsiella pneumoniae*, *Pseudomonas aeruginosa, Acinetobacter baumannii*, *Bacteroides* spp., *Clostridium* spp., etc. Over the past two decades, *16S rRNA* gene sequencing technology has been used for the identification and classification of bacterial species in many infection-associated diseases (Li et al., 2017, 2018; Rogers et al., 2017; Philips et al., 2019) and microbiota-associated pancreatic diseases (Li et al., 2017; Rogers et al., 2017). The characteristic of bacteremia in AP patients could be defined and analyzed using polymerase chain reaction (PCR) (de Madaria, 2005; Bhutta and Ashley, 2013) or denaturing gradient gel electrophoresis (DGGE) (Li et al., 2013). The blood and neutrophil-associated microbiota (Li et al., 2018) as well as the gut microbiota associated with the progression of SAP (Philips et al., 2019) can be determined and analyzed using *16S rRNA* gene next-generation sequencing (NGS). However, the studies on SAP-and CAP-related gut microbiota, especially those on pancreas-associated infections, are limited. Moreover, the correlation of microbial diversity in the pancreas with the severity of AP also remains unclear. Furthermore, *16S rRNA* gene NGS has high sensitivity and specificity in identifying the composition of the microbiota, especially the anaerobic, low-abundant, fastidious, and/or slow-growing microbiota, thereby effectively implementing intervention measures to achieve better clinical outcomes (Dyrhovden et al., 2019, 2020; Stebner et al., 2021).

This study aimed to identify the composition of microbiota in the infected pancreatic fluids obtained from SAP and CAP patients using *16S rRNA* gene NGS and further analyzed their source. The results were further evaluated by comparing them to those obtained from aerobic cultures.

## 2. Materials and methods

### 2.1. Patients and clinical specimens

In this study, 56 patients, who were admitted to the general surgery department of Jinling Hospital in Nanjing, Jiangsu Province, China, from June 2020 to May 2021 and diagnosed with SAP or CAP based on the presence of organ failure and/or local determinants, were enrolled. A total of 78 pancreatic fluid samples were collected from percutaneous catheter drainage or puncture using a 10 mL tube, immediately transported to the microbiological laboratory for the detection and identification of bacteria, and then stored at −80°C. From these patients, one pancreatic fluid sample was collected from each of 41 patients, 2 samples were collected from each of 10 patients, 3 samples were collected from each of 3 patients, and 4 were collected from each of 2 patients. The demographic and clinical characteristics of all the patients were collected from the electronic medical records of the hospital and summarized.

### 2.2. Routine culture

A drop of pancreatic fluid was taken for smear microscopy. A 50 μL sample from each pancreatic fluid sample was then inoculated onto the plates of blood agar, chocolate agar, and MacConkey agar (Thermo Fisher Scientific Inc., Shanghai, China), respectively. The plates were incubated at 35°C in a 5% CO_2_ atmosphere and observed after 72 h. Several colonies were selected by observation through the naked eye. Gram staining was performed based on the standard operation protocol of our laboratory, and biochemical identification was performed using VITEK^®^ 2 COMPACT (bioMérieux, Marcy-l’Étoile, France). Anaerobic culturing was not performed because the samples from surgery or drainage did not guarantee an anaerobic environment.

### 2.3. Sample processing and DNA extraction

A 500-μl pancreatic fluid sample was centrifuged at 14,000 g for 10 min, and the supernatant was removed. Then, two 3 mm nickel beads were added to each sample tube and shaken at 3,000 rpm for 5 min. Finally, DNA was extracted using the Qiagen DNA mini kit (Qiagen, Shanghai, China) following the manufacturer’s instructions, eluted with 100 μL distilled water, and stored at −80°C.

### 2.4. Library preparation and sequencing

The V3–V4 region of the *16S rRNA* gene was amplified using universal primers (341F, 5′-CCTAYGGGRBGCASCAG-3′ and 806R, 5′-GGACTACNNGGGTATCTAAT-3′), obtaining a product with approximately 470 bp length. PCR was performed with Phusion^®^ High-Fidelity PCR Master Mix (New England Biolabs, Ipswich, Massachusetts, United States) reagent using a thermal cycler (Axygen MaxyGene II, Axygen Scientific, Union City, United States). The reaction mixture contained 0.2 μM of each primer, 1× Phusion Master Mix, and 10 ng DNA template. The PCR reaction conditions were as follows: initial denaturation at 98°C for 1 min; 30 cycles of denaturation at 98°C for 10 s, annealing at 50°C for 30 s, and extension at 70°C for 5 min; and final extension at 70°C for 5 min.

The PCR products were analyzed using agarose gel electrophoresis and purified using GeneJET NGS Cleanup Kit (Thermo Fisher Scientific Inc., Shanghai, China). Library preparation was performed using the TruSeq^®^ DNA PCR-Free Sample Preparation Kit (Illumina, Redwood City, CA, United States) following the manufacturer’s instructions. The library preparation was assessed using Qubit@ 2.0 Fluorometer (Thermo Fisher Scientific Inc., United States) and Agilent Bioanalyzer 2100 system (Agilent Technologies Inc., Santa Clara, CA, United States). Finally, the pooled libraries were diluted and sequenced using the NovaSeq platform, and 2 × 250 paired-end raw sequence reads were obtained. Following the processing of each clinical sample, the sterile deionized water as a negative control was processed in parallel.

### 2.5. Data analysis

The quality of sequence reads was assessed using Fastp (version 0.23.2) (Chen et al., 2018), and the low-quality reads were removed. Using VSEARCH (v2.18.0) (Rognes et al., 2016), the paired sequence reads were merged after trimming the primers, and the fragments shorter than 250 bp were filtered out. Operational taxonomic unit (OTU) clustering was performed using VSEARCH with a 100% similarity. The sequences were annotated using the Basic Local Alignment Search Tool (BLAST, version 2.12.0+) against the National Center for Biotechnology Information (NCBI) *16S rRNA* database (https://ftp.ncbi.nlm.nih.gov/blast/db/16S_ribosomal_RNA.tar.gz, updated at 2022-01-08) (Camacho et al., 2009). Furthermore, each detection described as pathogens in the previous study or considered to be clinically relevant by the clinician were considered to be clinically reporting pathogens. Importantly, the detections considered to be contaminants, colonizers, and commensals were excluded.

### 2.6. Statistical analysis

All the data were expressed as the mean and standard deviation of the mean or as median and range between brackets as applicable. All the statistical analyses were performed using the R package (version 4.0.2) and the vegan package (version 2.5-7). The significance level was set at *p*-value <0.05. Alpha diversity was analyzed using Shannon’s diversity index to describe the diversity of bacterial species in each sample. Shannon’s diversity indices of SAP and CAP were compared using Welch’s *t*-test. Bray–Curtis distance algorithm with an unweighted pair-group method and arithmetic means was used for the hierarchical clustering analysis of bacterial diversity in the SAP and CAP patients. Principle coordinate analysis (PCoA) was performed on the Bray-Curtis distance to analyze the differences in the bacterial compositions under various factors, such as local complications, organ failure, etc. The analysis of similarities (ANOSIM) test was used to compare the differences in bacterial composition between the patients with SAP and CAP.

## 3. Results

### 3.1. Clinical characteristics of patients

**Figure 1:**
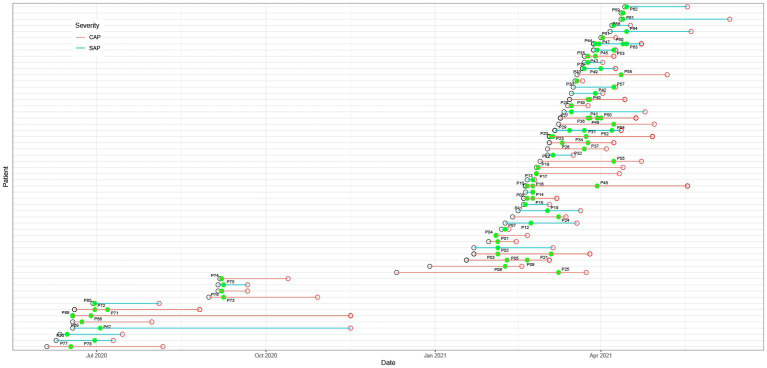
Hospitalization schedule, including hospital admission (black hollow circle), discharge (red hollow circle), and sampling (green dot). The green and red lines represent severe acute pancreatitis (SAP) and critical acute pancreatitis (CAP), respectively.

As listed in Table 1, a total of 56 patients, including 39 males and 17 females, with a mean age of 42 were enrolled in this study. Except for 2 recovering patients with SAP, all the other SAP and CAP patients were transferred from lower-level hospitals to Jinling Hospital. Based on the AP severity, the patients were divided into SAP (*n* = 26) and CAP (*n* = 30) patients. The results of statistical analysis showed that age and gender had no significant effect on the grouping. The mean durations of intensive care unit (ICU) stay were 26 days for SAP patients and 46 days for CAP patients. The CAP patients had significantly longer ICU stays than the SAP patients (*p* < 0.05). Most pancreatic fluid samples were obtained within 1–2 days after admission to the hospital (Figure 1). The etiology of AP was categorized as alcoholic, biliary, hyperlipidemia, endoscopic retrograde cholangio-pancreatography (ECRP), and unknown cause. Finally, 48 patients significantly improved and were transferred to the general ward.

**Table 1:** Demographic and clinical characteristics of all the patients.

Number of patients	56
Mean age (SD; min–max), year	42 (13; 13–68)
Gender, male	39 (69.64%)
Smoking	10 (17.86%)
History of alcoholism	17 (30.36%)
ICU stays (SD; min–max), days	37.79 (32; 2–197)
Improvement	48 (85.71%)
Severity	
Severe	26 (42.31%)
Critical	30 (57.69%)
CRP (SD; min–max)	112.37 (70.97; 0.45–385)
PCT (SD; min–max)	2.93 (6.62; 0.029–46.53)
Etiology	
Alcoholic	2 (3.57%)
Biliary	22 (39.29%)
ERCP	1 (1.79%)
Hyperlipidemia	25 (44.64%)
Unknown cause	6 (10.71%)
Local complications	
Acute necrotic collection	8 (14.29%)
Infected pancreatic necrosis	47 (83.93%)
Walled-off necrosis	1 (1.79%)
Systematic complications	
ARDS	37 (66.07%)
AKI	21 (37.5%)
Shock	7 (12.5%)
Sepsis	9 (16.07%)
Liver damage	4 (7.14%)

CRP, C-reaction protein; PCT, procalcitonin; ERCP, endoscopic retrograde cholangio-pancreatography; ARDS, acute respiratory distress syndrome; AKI, acute kidney injury.

### 3.2. Workflow of *16S rRNA* gene NGS

Of the total 78 pancreatic fluid samples obtained from the patients, 56 samples including 52 aerobic culture-positive and 4 aerobic culture-negative samples, had PCR products of expected length and were sequenced. Sequencing was abandoned for 19 aerobic culture-negative samples because the PCR products of the expected length were not obtained. Three samples with no PCR products, as well as the sterile deionized water, were used as negative controls. The number of mean raw reads was 199,252. After quality control using Fastp, a total of 198,383 reads were reserved. For the two negative controls, the mean raw reads were 6,877, among which, 6,864 were left after quality control. Using VSEARCH, a total of 492,528 OTUs clusters with 100% similarity were obtained. The clusters were aligned against the NCBI *16S rRNA* database using BLAST with a threshold of 97% similarity. The results showed that a total of 381,718 OTUs were annotated. In the negative controls, *Romeboutsia timonensis* (84 reads) and *Akermansia muciniphila* (64 reads) had the highest number of reads. Therefore, a cut-off value of 100 reads was set, and the species emerged in negative controls as background contaminants were removed. The isolation sources of identified species were analyzed using BacDive^1^ (Reimer et al., 2019), and a reference strain was selected when there were multiple strains in the database.

### 3.3. Composition of bacterial species identified using culturing and *16S rRNA* gene NGS

**Figure 2:**
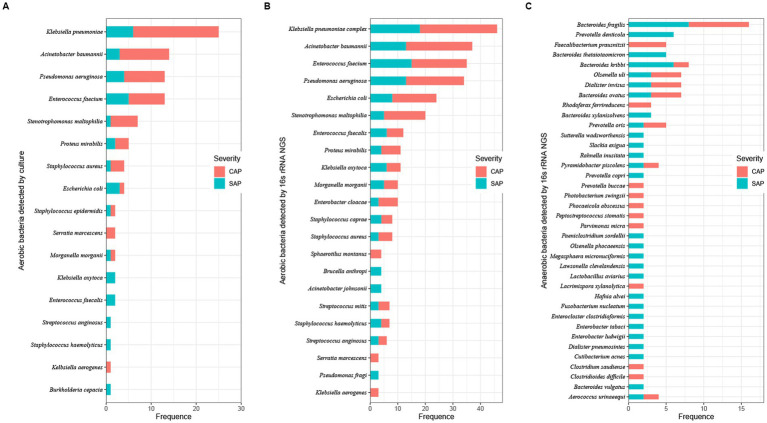
Number of bacterial taxa in pancreatic fluids identified using aerobic culturing and *16S rRNA* gene NGS. Bacterial species were identified using aerobic culturing **(A)** and using the *16S rRNA* gene NGS (aerobes: **B**; anaerobes: **C**). Colors represent the severity of AP (red, CAP; light blue, SAP).

**Figure 3:**
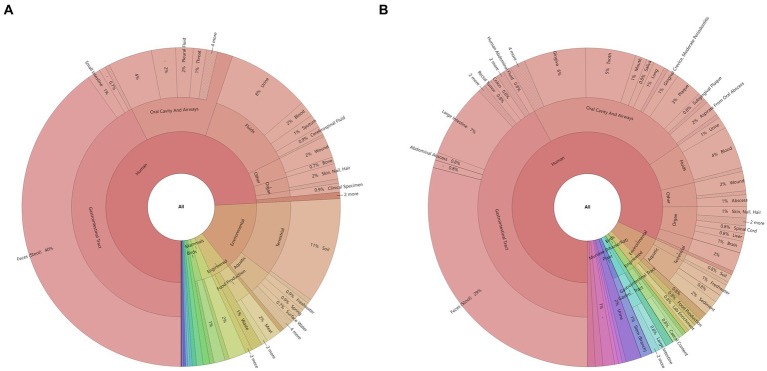
Identification of bacterial sources in the pancreatic fluid samples using the BacDive database: **(A)** aerobes and **(B)** anaerobes. The isolation sources of bacteria identified using the *16S rRNA* gene NGS were queried using the prokaryotic meta-database BacDive (see footnote 1). The reference strain was selected when multiple strains were present in the database.

Twenty-six of the total 78 samples were aerobic culture-negative, while 99 aerobic cultures were isolated from the remaining 52 samples, which belonged to 17 species in 11 genera (Figure 2A and Supplementary Table S1). Among these species, *K. pneumoniae* complex was identified in 48.08% (25/52) of the samples. The *16S rRNA* gene NGS detected a total of 660 bacteria in 56 samples (52 aerobic culture-positive and 4 aerobic culture-negative samples; Figures 2B,C and Supplementary Table S1), which belonged to 218 species in 123 genera, including 91 aerobic species in 75 genera and 127 anaerobic species in 48 genera. Among the aerobic bacterial species, *K. pneumoniae* complex (46/56), *Acinetobacter baumannii* (37/56), *Enterococcus faecium* (35/56), *Pseudomonas aeruginosa* (34/56), *Escherichia coli* (24/56), and *Stenotrophomonas maltophilia* (20/56) were the predominant species. Among the anaerobic bacterial species, *B. fragilis* (16/56), *B. kribbi* (8/56), *B. ovatus* (7/56), *Dialister invisus* (7/56), and *Olsenella uli* (7/56) were the predominant species. Additionally, polymicrobial infections were found in 54 samples (98.21%), and only four samples had a mono-bacterial infection of *Enterococcus faecium* (sample P18, 189 reads, 51.78%), *A. baumannii* (sample P68, 120,432 reads, 99.99%), *K. pneumoniae* complex (sample P73, 13,298 reads, 99.97%), or *K. pneumoniae* complex (sample P74, 38,037 reads, 99.99%). Using the prokaryotic meta-database BacDive (see footnote 1), the isolation sources of pancreatic bacterial infections were identified as intestines (aerobes 43%; anaerobes 43%) and oral cavity and airways (aerobes 17%; anaerobes 24%; Figure 3).

*16S rRNA* gene NGS identified 96 bacterial species, which were also identified by aerobic culturing (Supplementary Table S1). *Stenotrophomonas maltophilia* (sample P17, 10 reads), *Pseudomonas aeruginosa* (sample P25, 81 reads), and *Escherichia coli* (sample P62, undetermined) had less than 100 reads and were considered undetermined. Among the 56 samples, *16S rRNA* gene NGS identified 660 bacterial strains, including 450 aerobic and 256 anaerobic bacterial species, while aerobic culturing identified 21.33% (96/450, excluding 3 strains, which were undetermined or had less than 100 reads).

### 3.4. Dynamic changes in bacterial infection in patients with SAP or CAP

**Figure 4:**
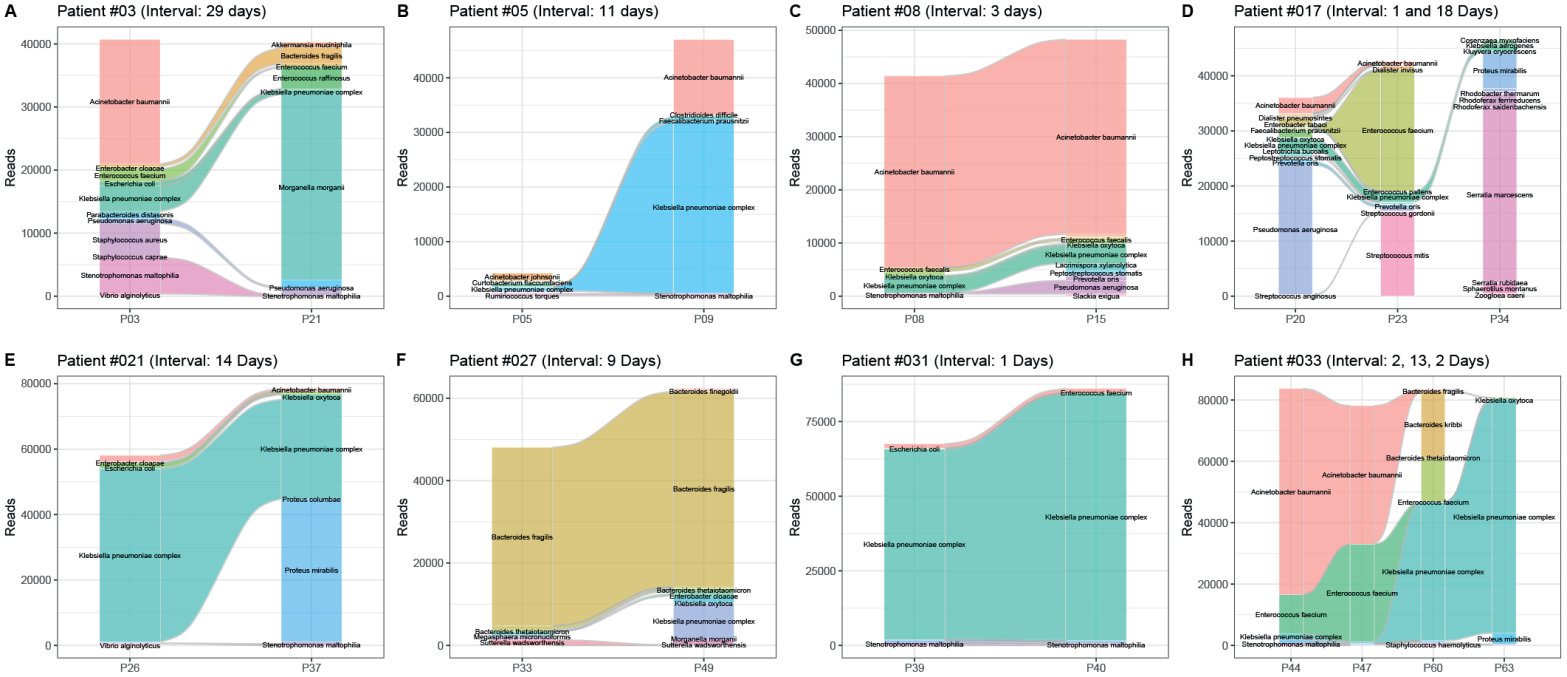
Dynamic changes in the bacterial profile and abundance in 8 patients with pancreatitis. Patient numbers and sampling intervals are marked. **(A)** Patient #03, sampling interval 29 days; **(B)** patient #05, sampling interval 11 days; **(C)** patient #08, sampling interval 3 days; **(D)** patient #017, sampling interval 1 and 18 days; **(E)** patient #21, sampling interval 14 days; **(F)** patient #027, sampling interval 9 days; **(G)** patient #031, two sampling interval 1 day each; **(H)** patient #033, sampling intervals 2, 13, and 2 days.

Among 56 patients, multiple pancreatic fluid samples were collected from 15 patients with an average sampling interval of 7.4 days. The dynamic changes in the bacterial profile and abundance in pancreatic infection were analyzed using the *16S rRNA* gene NGS (Figure 4). When the sampling interval was less than 2 weeks, the composition of bacterial species did not change much at the pancreatic site. On the other hand, when the sampling interval was more than 2 weeks later, the bacterial species in the pancreatic fluid changed greatly, which was consistent with the results of the aerobic culture. Importantly, bacteria with low abundance were often ignored in the early stage due to the limitation of culture and could become the main pathogen in the later stage (Figures 4B,H).

### 3.5. Comparative analysis of bacterial composition based on AP severity

**Figure 5:**
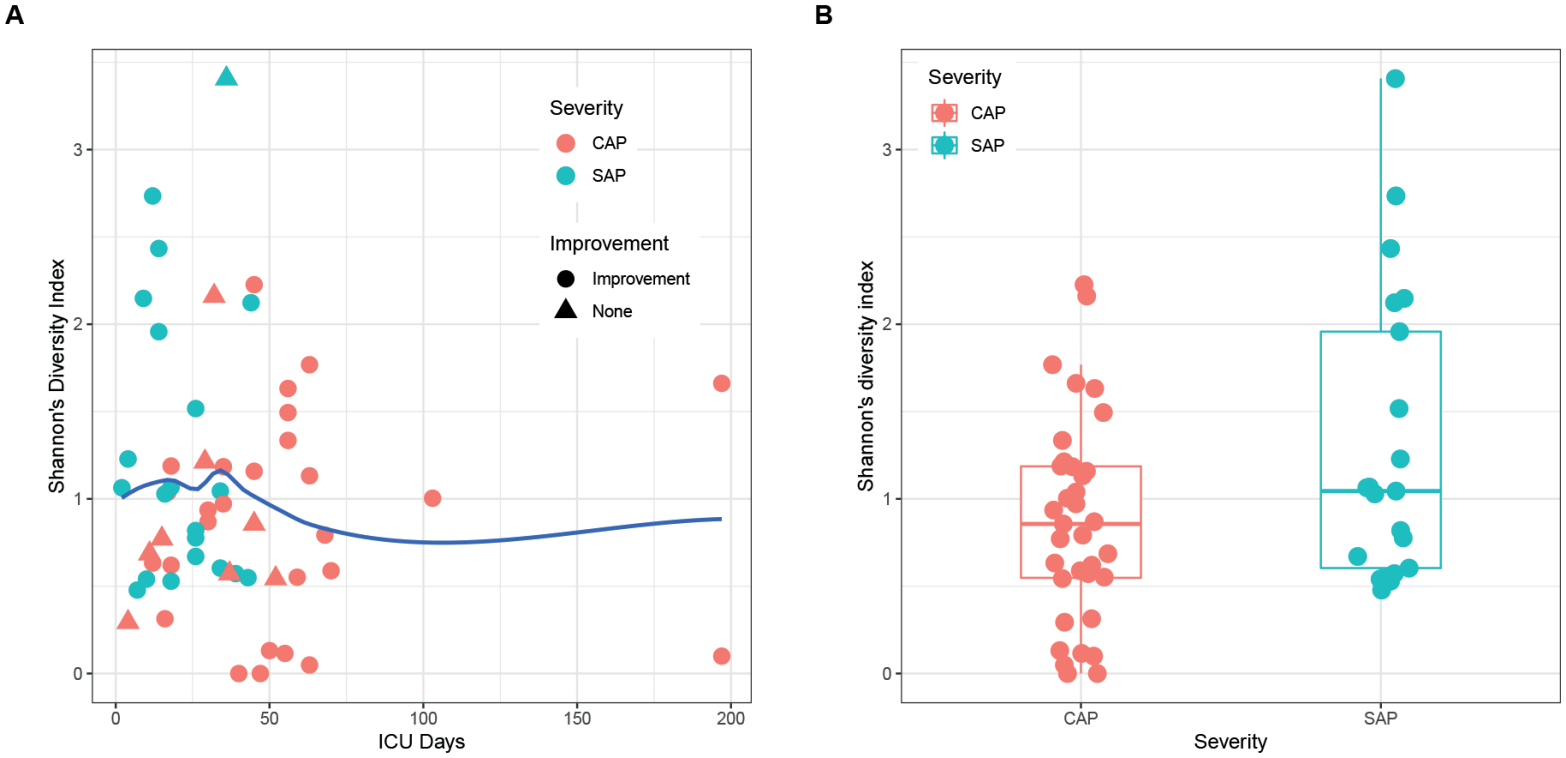
**(A)** Effects of ICU stay duration and improvement of patients on Shannon’s diversity indices. The local regression fitting line was plotted using ggplot2 (blue line), and the dots represent the improvement of patients. **(B)** Comparison of beta diversity between SAP (light blue) and CAP (red).

**Figure 6:**
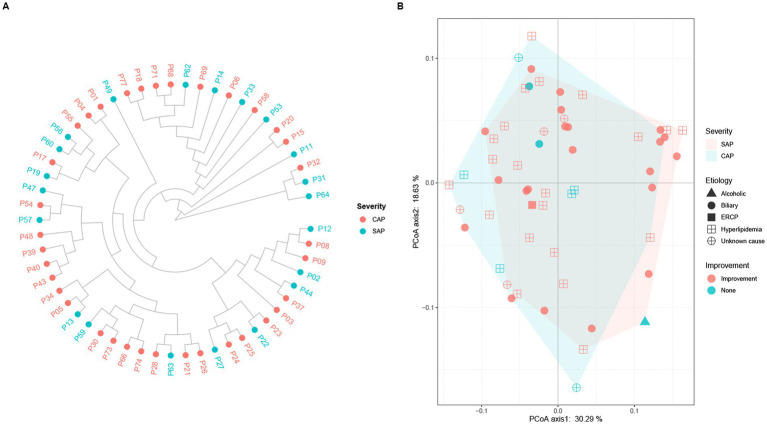
**(A)** Hierarchical clustering and **(B)** principal coordinate analysis (PCoA) of pancreatic fluid samples obtained from the SAP and CAP patients based on Bray–Curtis dissimilarity. **(A)** Each dot represents one pancreatic fluid sample (red, CAP; light blue, SAP). There is no clear clustering into separate groups in the comparative analysis of AP severity. **(B)** Each dot represents one sample, and the characteristics of each patient including etiology (alcoholic, biliary, ECRP, hyperlipidemia, and unknown cause) and outcome (improvement, and none) are shown. There is a large overlapping between the light red area representing SAP and the light blue area representing CAP.

The alpha diversity analysis of 56 pancreatic fluid samples (Figure 5) using Shannon’s diversity index showed that the alpha diversity did not change significantly with the increase in the duration of ICU stay (Figure 5A). Welch’s *t*-test also showed that there was no significant difference between SAP and CAP (*p* = 0.05036, Figure 5B). Furthermore, there was no significant difference in Shannon’s diversity indices between the CAP patients who did not achieve significant treatment effects and those who achieved significant improvement. The hierarchical clustering of these samples using an unweighted pair-group method with arithmetic means (UPGMA) indicated that these samples had a similar bacterial composition (Figure 6A). PCoA was performed based on the distance matrix of Bray–Curtis dissimilarity. As shown in Figure 6B, there was no clear divergence trend in beta diversities of SAP and CAP, while considering other factors, such as etiology, prognosis, etc. The result of ANOSIM also showed no significant difference between the two groups (*R* = 0.03005, *p* > 0.05). In addition, the relative abundances of common species, such as *A. baumannii*, *Pseudomonas aeruginosa*, *K. pneumoniae* complex, and *S. maltophila*, were much higher in CAP patients than those in the SAP patients, which were mostly multidrug-resistant nosocomial bacterial species.

## 4. Discussion

Culture is a routine detection method for the identification of microbial species in clinical microbiological laboratories. However, it must be ensured that the microorganisms are culturable and can grow on solid or liquid media *in-vitro*. Furthermore, most clinical microbiology laboratories cannot perform anaerobic culturing due to the limitation of an oxygen-free environment, and the cultures are identified using subjective and random morphological identification methods for polymicrobial infection. As a novel method, *16S rRNA* gene NGS has great advantages as compared to culture, especially for the identification of anaerobic, low-abundant, fastidious, and/or slow-growing microorganisms. Infection is one of the risk factors, contributing to the high mortality of AP (Petrov et al., 2010; Bhutta and Ashley, 2013). The detection and identification of bacterial composition are of great significance to implement requisite interventions for AP (Mourad et al., 2017; Ning et al., 2019). This study used *16S rRNA* gene NGS to identify the bacterial composition in 78 pancreatic fluid samples in comparison to culturing. In the 52 samples, aerobic culturing identified 99 bacterial specimens, among which, *16S rRNA* gene NGS identified 96 bacterial specimens. Three patients had positive aerobic cultures with discordant *16S rRNA* gene sequencing results. This might be due to the low abundance of positive aerobic cultures and presence of more abundant bacteria in the samples, which might have interfered with the amplification and sequencing of the *16S rRNA* gene (Fida et al., 2021).

OTUs were clustered using VSEARCH and aligned with the NCBI *16S rRNA* database using BLAST in this study. Almost all the *P. aeruginosa* species were lost in clustering with 97% similarity as a threshold. A high clustering threshold might be necessary for the detection and identification of pathogenic bacteria using *16S rRNA* gene NGS, especially when multiple infections are suspected, which is different from microbiome analysis (Ikegami et al., 2021). Therefore, 100% similarity was set as a threshold. There are often DNA contaminations in the *16S rRNA* gene NGS. Dyrhovden et al. (2019, 2020, 2021) proposed a criterion for setting the threshold with the most abundant pollutant species in negative controls. Sterilized deionized water and culture-negative pancreatic fluid sample with no PCR products, was used as negative controls, and the bacterial composition was determined using the *16S rRNA* gene NGS. The number of reads with the highest abundance in negative controls was set as the threshold. In the two negative controls, *Romeboutsia timonensis* and *Akermansia muciniphila* with 84 and 64 reads were detected, respectively. Therefore, 100 reads were set as the threshold, which was slightly more than the reads of the most abundant species in negative controls. However, this might have led to ignoring some low-abundance species, such as *S. maltophilia* (10 reads) in sample P17 and *P. aeruginosa* (81 reads) in sample P25.

Bacterial translocation is an important mechanism of AP infection. A previous study showed that the predominant aerobic bacteria included *E. coli*, *K. pneumoniae*, *streptococcus* group D, and *S. aureus*, while the predominant anaerobic bacteria included *Peptostreptococcus* species, *B. fragilis* group organisms, *Clostridium* species, *Prevotella* species, *Veillonella* species, and *Fusobacterium* species (Brook and Frazier, 1996). In the current study, the results showed that the predominant aerobic bacteria were *K. pneumoniae* complex, *A. baumannii*, *E. faecium*, *P. aeruginosa*, and *E. coli*, while the predominant anaerobic bacteria were *B. fragilis*, *B. kribbi*, *B. ovatus*, *D. invisus*, and *O. uli*. The main pathogens responsible for CAP and SAP have changed somewhat as compared to those reported in previous studies. This might be related to the severity or antibiotic use in the last two decades. Approximately 42% of aerobic bacteria and 43% of anaerobic bacteria might be derived from the gut, which was different from the bacterial communities present in blood-related infections (de Madaria, 2005; Li et al., 2013, 2018). Moreover, the current study also indicated that the source of a considerable proportion of bacteria was oral and airway microbiota as well as from the environment, which was similar to the results of the pancreas-related microbial communities (Li et al., 2017). This might due to the collection of samples from the SAP and CAP patients, who were affected by long-term hospitalization, such as antibiotics, surgery, etc., and were immunocompromised, resulting in infections from various sources. Disinfection of the hospital environment and oral cleaning are necessary to prevent bacteria entering the pancreas through the gastrointestinal tract or surgical incisions from oral and environmental sources. The changes in bacterial profile and abundance in pancreatic samples were dynamically analyzed using the *16S rRNA* gene NGS. The results showed that the abundance of different bacteria varied greatly, and some low-abundance species were easily overlooked. The colonized or commensal bacteria usually do not exist in the pancreas. Some bacteria originating from the gut, respiratory tract, or environment, might appear in the pancreas and are not necessarily considered pathogenic (Blauwkamp et al., 2019; Miller et al., 2019; Fida et al., 2021; Gu et al., 2021). It is necessary to make a comprehensive and available diagnosis by combining the NGS results, clinical signs of patients, and other laboratory tests.

There were certain limitations to this study. First, all the patients were referred from other hospitals, and their previous treatment courses were unknown, while the anaerobic culturing was also not performed. Second, since all the specimens in this study came from one hospital, a larger-scale multi-center study is needed to explore the sources of all infections in order to clearly determine the type of bacterial infection in AP patients. Finally, there might be some errors in the isolation sources of bacteria; for example, although *E. coli* is generally believed to be originated from the intestine, it also appears in urinary tract infections.

In conclusion, in this study, a total of 78 pancreatic fluid samples were collected from 56 SAP and CAP patients, and the compositions of their bacterial profiles were determined using *the 16S rRNA* gene NGS. The results showed that the predominant aerobic bacteria were *K. pneumoniae*, *A. baumannii*, *E. faecium*, *P. aeruginosa*, *E. coli*, etc., while the predominant anaerobic bacteria were *B. fragilis*, *B. kribbi*, *B. ovatus*, *D. invisus*, and *O. uli*, etc. As compared to aerobic culturing, *16S rRNA* gene NGS might detect more bacteria; however, contamination and data analysis might limit its results. *16S rRNA* gene NGS can be used as an effective supplement to culturing.

## Data availability statement

The datasets presented in this study can be found in online repositories. The names of the repository/repositories and accession number(s) can be found at: https://www.cncb.ac.cn/, PRJCA015500.

## Ethics statement

The studies involving human participants were reviewed and approved by 2016NZGKJ-049. Written informed consent to participate in this study was provided by the participants’ legal guardian/next of kin.

## Author contributions

NS and XL: study design. YC, JZ, JC, and NS: data analysis. XL and NS: funding acquisition. YC, HH, and JC: resources. NS, YC, WG, and JW: experimental studies. XL: supervision. NS: writing-original draft. All authors contributed to the article and approved the submitted version.

## Funding

This study was funded by the Suqian Sci&Tech Program (SY202214), National Natural Science Foundation of China (no. 81601857), and the Health Technology Development Special Foundation of Nanjing City (no. YKK18216).

## Conflict of interest

The authors declare that the research was conducted in the absence of any commercial or financial relationships that could be construed as a potential conflict of interest.

## Publisher’s note

All claims expressed in this article are solely those of the authors and do not necessarily represent those of their affiliated organizations, or those of the publisher, the editors and the reviewers. Any product that may be evaluated in this article, or claim that may be made by its manufacturer, is not guaranteed or endorsed by the publisher.

## Footnotes

^1^ https://bacdive.dsmz.de/
